# Antioxidant Effect of Alpha-Lipoic Acid in 6-Hydroxydopamine Unilateral Intrastriatal Injected Rats

**DOI:** 10.3390/antiox9020122

**Published:** 2020-02-01

**Authors:** Pavlina Andreeva-Gateva, Lubomir Traikov, Zafer Sabit, Dimitar Bakalov, Radka Tafradjiiska-Hadjiolova

**Affiliations:** 1 Department of Pharmacology and Toxicology, Faculty of Medicine, Medical University-Sofia, 1431 Sofia, Bulgaria; 2 Department of Medical Physics and Biophysics, Faculty of Medicine, Medical University-Sofia, 1431 Sofia, Bulgaria; 3 Department of Pathophysiology, Faculty of Medicine, Medical University-Sofia, 1431 Sofia, Bulgaria

**Keywords:** alpha-lipoic acid, oxidative stress, 6-hydroxydopamine, glutathione peroxidase, lipid peroxidation, Parkinson’s disease

## Abstract

The toxin 6-hydroxydopamine (6-OHDA) is a highly oxidizable dopamine (DA) analog that is widely used for reproducing several cell processes identified in Parkinson’s disease (PD). Due to the close similarity of its neurotoxic mechanism to those of DA, it is suitable as a model for testing the effects of potentially neuroprotective drugs. This study aimed to evaluate the effect of alpha-lipoic acid (LA) on brain oxidative stress (OS) in unilateral intrastriatal (6-OHDA) injected rats. Forty male Wistar rats, four months old (220–260 g), were evaluated. Half of them received LA (35 mg/kg i.p.) from the start to the end of the experiment. On day 2 of the trial, ten LA-supplemented rats and ten non-LA-supplemented rats were subjected to the apomorphine test. Brain homogenates were evaluated for thiobarbituric acid-reactive substances (TBARS) and glutathione peroxidase (GPx) activity. The same evaluation procedures were repeated on day 14 with the remaining animals. An increased TBARS level and decreased GPx activity, suggestive for OS, were recorded in homogenates on day 14 vs. day 2 of the experiment in the 6-OHDA treated rats. The simultaneous application of LA mitigated these changes. Our study demonstrates that the low dose of LA could be of value for decreasing the OS of the neurotoxic 6-OHDA, supporting the need for further studies of the benefit of LA treatment in PD.

## 1. Introduction

Parkinson’s disease (PD) is one of the most common neurodegenerative disorders, with an incidence of about 1–1.5% of the population over the age of 60. It is primarily associated with the loss of dopamine (DA)-producing neurons in the substantia nigra and with Lewy bodies, but its symptomatology is not restricted to the motor features. Extensive regions of the nervous system, various neurotransmitters, and protein aggregates other than Lewy bodies are implicated in the pathology of PD [1]. A complex interplay between genetics and environmental factors results in an increase in oxidative stress (OS), as well as inflammation, autophagy, apoptosis, protein aggregation, and changes in the levels of neurotransmitters [2].

The role of OS seems to be an essential one for the pathogenesis of PD. OS results from an imbalance between production and detoxification of reactive oxygen species (ROS). Dopaminergic neurons in the substantia nigra present high baseline ROS levels and low activity of glutathione peroxidases (GPx) (EC 1.11.1.9), a family that comprises eight isoforms involved in H_2_O_2_ detoxification. The protective antidegenerative role of sulfhydryl antioxidants in the striatum was attributed to its capacity to neutralize H_2_O_2_ in vivo [3]. GPx can use γ-glutamylcysteine as a reductant for H_2_O_2_ [4]. For the brain, the critical activity is attributed to the GPx4 isoform, as tamoxifen-induced Gpx4 ablation in mice results in a lethal phenotype with neuronal loss [5]. A newly identified pathway of cell death, referred to as ferroptosis, has recently been linked to both GPx4, and PD [6]. Ferroptosis refers to an iron-dependent cell death pathway that involves the depletion of intracellular reduced-glutathione (GSH) levels. The apoptotic signaling pathway, protein kinase phosphorylation, and oxidant-mediated activation of nuclear factor kappa B (NFκB) all are processes critically dependent on intracellular and tissue GPx [7].

Due to their long axons and millions of synapses, which need high energy to work properly, dopaminergic neurons in the substantia nigra are particularly vulnerable to OS [8]. There is also an abundance of ROS-generating enzymes, such as tyrosine hydroxylase (EC 1.14.16.2) and monoamine oxidase (EC 1.4.3.4). DA is easily oxidized both spontaneously and enzymatically to produce DA quinone species, which are able to covalently modify cellular nucleophiles, such as GSH, proteins cysteinyl residues, α-synuclein, parkin and other proteins, which are all implicated in the pathophysiology of PD [9]. DA easily autooxidates and converts to neuromelanin, which promotes the formation of ROS. A self-perpetuating vicious cycle of neurodegeneration ensues [10]. The mitochondrion is one of the main sites of ROS production. It is also a target of ROS-induced damage. Protein folding in the endoplasmic reticulum and cellular calcium load are other relevant sources of ROS.

Neuroinflammation due to an overactivated and/or chronically activated state of microglia is another source of excessive and uncontrolled release of free radicals. Molecular mechanisms underlying these pathophysiological processes are still under evaluation, but recent evidence comes from studies on high-mobility group box 1 (HMGB1) [11], mammalian target of rapamycin (mTOR) signaling [12], and the nuclear factor, erythroid 2 like 2 (Nrf2) [13], among others. Recent breakthroughs from genetic studies revealed that lysosomal system dysfunction is one of the earliest pathogenetic mechanisms. These changes go in parallel with the other pathogenetic mechanisms of PD [14]. Products of lipid peroxidation can support the formation of toxic soluble oligomers in a dose-dependent manner. Hence, prevention of lipid peroxidation can inhibit the abnormal aggregation of α-synuclein, and thereby delay the pathogenesis of PD [15].

Autophagy and OS are mutually interdependent. OS may affect autophagy through many mechanisms, i.e., activation of adenosine monophosphate-dependent protein kinase (AMPK, EC 2.7.11.11) [16], inhibition of the mTOR signaling pathway [17], or activation of the JNK pathway [18]. On the one hand, OS is a potent activator of autophagy; on the other hand, defective autophagy increases OS.

The approved therapy of PD only targets the amelioration of symptoms through the increase of the central effects of DA or blockade of acetylcholine. Mutations of the glucocerebrosidase gene were discovered as the most critical genetic vulnerability factor for PD, and drugs that interfere with this process are under investigation [19]. Unfortunately, even with that recent breakthrough in the PD treatment, the delay of the neurodegenerative process in PD by neuroprotective therapy is an important unmet clinical need. The central place occupied by OS both in PD progression and in complications from dopaminergic therapy (i.e., dyskinesia) imposes the question of the value of the use of antioxidants.

Alpha-lipoic acid (LA), also known as thioctic acid, is a naturally occurring compound. Since the discovery of its antioxidant properties in the 1950s, LA has been widely studied for treating diabetic neuropathy, as an antioxidant and neuroprotective agent [20]. LA has the salubrious property of neutralizing free radicals without itself becoming one in the process [21]. Upon oxidation with ROS and prooxidants, LA may be reconstituted from dihydrolipoic acid and other reduced forms [22]. Dihydrolipoic acid gained much interest as a scavenger for superoxide and peroxyl radicals and as a facilitator of the recycling of vitamin E [23]. Moreover, LA has a potential role as a chelator of metals. It also restores the normal levels of intracellular GSH and ascorbic acid through nonenzymatic regeneration after depletion caused by toxicants, environmental pollutants, or senescence [24]. LA is a direct and indirect antioxidant, which is able to increase the expression of antioxidant enzymes and regulate the expression of proteins involved in energy metabolism, mitochondrial biogenesis, and structural integrity of mitochondria. All of these effects are mediated both by the antioxidant response element (ARE; Keap/Nrf2 pathway) and by the peroxisome proliferator-activated receptor-gamma co-activator-1 alpha (PGC-1 alpha) [25]. 

LA has been considered a powerful micronutrient that manifests a range of pharmacological properties. It optimizes carbohydrate and fatty acid utilization and thus supports energy metabolism. Additionally, LA assists electron transport and adenosine triphosphate synthesis, decreasing oxidative and nitrosative stress, counteracts protein misfolding, inflammation stimuli, iron, and various environmental and endogen toxins. Meta-analyses indicate some beneficial effects on glycemic and inflammatory biomarkers [26,27], and lipid profile improvement [28]. In the field of neurology, clinical evidence exists mainly for the treatment of diabetic polyneuropathy [29]. 

Many aspects of the LA-effect, however, still need to be clarified, especially its application in the treatment and prevention of neurodegeneration [30]. There are some contradictions in the results of LA testing in 6-OHDA-treated models. As an example, in vitro studies aiming to explain the neuroprotective mechanisms of LA demonstrated either activation [31] or inhibition [32] of autophagy. LA demonstrated anti-inflammatory properties and promoted increases in intracellular GSH formation, which has been postulated to be beneficial in neurodegenerative disease [33]. On the other hand, the effect of LA on the GSH level seems controversial, as earlier research failed to confirm GSH restoration or protection against 6-OHDA toxicity [34]. Our study aims to evaluate the effect of LA on brain OS in 6-OHDA unilateral intrastriatal injected rats.

## 2. Materials and Methods 

### 2.1. Drugs and Chemicals

We used (R)-(+)-α-lipoic acid (analytical standard, №04471, Supelco, Sigma-Aldrich, Bellefonte, PA, USA) for all experiments. Phosphate buffer, 6-OHDA, desipramine, ascorbic acid, phosphate-buffered saline (PBS), apomorphine, GPx activity kit (SAB2502102), and thiobarbituric acid-reactive substances (TBARS) kit (MAK085) were purchased from Sigma-Aldrich, USA. Ketamine hydrochloride was purchased from Richter Pharma, Austria. Xylazine was purchased from Alfasan International, the Netherlands. LA was kept light-protected, and LA-saline solutions were freshly prepared for the day. 

### 2.2. Design of the Study

**Figure 1:**
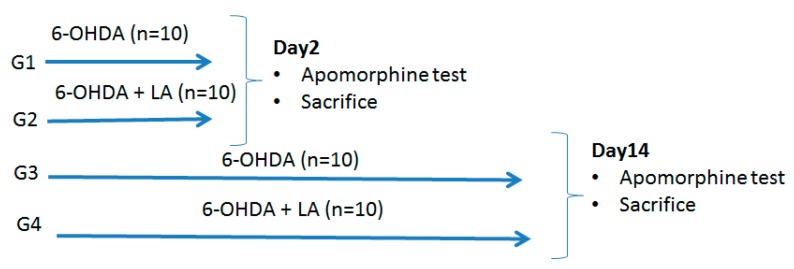
Flowchart of the study.

Forty male Wistar rats (220–260 g) were obtained from the vivarium of the Medical University of Sofia and housed under standard laboratory conditions (22 °C room temperature, 12 h light/12 h dark cycle), with *ad libitum* access to water and standard rat pellet diet. The experiment started one week after the habituation of the animals. At day one, all rats were randomly and equally distributed to four groups from G1 to G4, and all received a unilateral intrastriatal injection of 6-OHDA. The rats from the G2 and G4 groups received LA 35 mg/kg, i.p., dissolved in 1% Tween-80 solution daily, from the start to the end of the experiment. On day 2, the animals from G1 and G2 were subjected to an apomorphine test. After that, they were sacrificed with total brain removal for further evaluation. The same was done on day 14 of the experiment with the remaining rats from G3 and G4 groups. The flowchart of the study is presented in Figure 1.

### 2.3. Ethical Approval

The protocol of the study was approved by the Ethics Committee of the Bulgarian Food Safety Agency, Approval № 184/2017, and met the Animals in Research: Reporting In Vivo Experiments (ARRIVE) guidelines. 

### 2.4. 6-OHDA Unilateral Intrastriatal Injection

Thirty minutes before injection of 6-OHDA, the rats received the noradrenaline/serotonin reuptake inhibitor desipramine (25 mg/kg, i.p.) to prevent uptake of 6-OHDA by noradrenergic terminals and increase the selectivity of the neurotoxicity of the 6-OHDA. Standard stereotaxic procedures were used to target the medial forebrain bundle. Rats were anesthetized with an intramuscular injection of ketamine hydrochloride (80 mg/kg) and xylazine (10 mg/kg) and placed on a stereotaxic apparatus (Classic Lab Standard™ Stereotaxic Instrument, Stoelting, Dublin, Ireland). Then, 6-OHDA (12 μg in 3 μL saline with 0.2% ascorbic acid) was microinjected unilaterally (right side) into the medial forebrain bundle using the following coordinates (mm): from the bregma (anterior-posterior: −4.4; medial-lateral: +1.6; and dorsal-ventral: −8.0) [35]. Contralaterally (left side) phosphate buffer solution (PBS) was microinjected. The injection flow rate was 1 μL/min, using a 30-gauge cannula connected to a 10 μL Hamilton syringe. After the injection, 2 min of diffusion time was allowed before the gentle retraction of the cannula. 

### 2.5. Apomorphine Test

The apomorphine-induced turning of rats with a unilateral nigrostriatal lesion was observed after 2 mg/kg, i.p. [36]. The animals were allowed to habituate for 10 min in a quiet, isolated room and then placed in a transparent cylinder (diameter 33 cm and height 35 cm). The full rotations were counted for the first 60 min. Two observers independently recorded the number of rotations contralateral to the lesion of the nigrostriatal tract.

### 2.6. Brain Homogenate Handling

At day 2 and day 14, respectively, the animals were decapitated with a guillotine, without anesthesia, to avoid any possible biochemical changes due to the use of anesthetic agents. The brains were removed, flash-frozen in liquid nitrogen, and stored at −80 °C for further evaluation. Left and right lobes were separately processed. The brain tissues were weighed, placed on ice, and 10% w/v homogenates with PBS were prepared using a glass homogenizer. The homogenate was centrifuged at 4000× *g* at 4 °C for 10 min to yield a low-speed supernatant fraction for the TBARS and GPx assay.

### 2.7. GPx Activity Measurement

GPx activity was measured using a reaction coupled to glutathione reductase [37]. Reduced glutathione was used by GPx to reduce hydrogen peroxide (H_2_O_2_), which in turn was regenerated by the glutathione reductase from reduced glutathione and NAD(P)H. Results were expressed as enzyme activity in U/mg protein. One U of GPx represents 1 μmol NAD(P)H oxidized/min per mg protein.

### 2.8. TBARS Measurement

TBARS was assessed colorimetrically [38]. Tissue homogenate was treated with 10% trichloroacetic acid and centrifuged at 1600× *g* for 15 min at 4 °C. The supernatant was incubated with 0.67% thiobarbituric acid for 10 min at 100 °C. In parallel, a standard curve was constructed with malondialdehyde and determined at 532 nm. The concentration of lipid peroxidation products was calculated and expressed as malondialdehyde. TBARS were expressed as nmol/mg tissue protein.

### 2.9. Protein Measurement

Protein was determined according to the method of Lowry et al. (1951), with bovine serum albumin as standard [39]. 

All laboratory evaluations were performed with the spectrophotometer S-220 UV/Vis, Boeco, Germany.

### 2.10. Statistics

All data are presented as mean values (M) and standard deviations (SD). For the statistical assessment of the results from the Apomorphine test, the nonparametric Kruskal–Wallis one-way analysis of variance (ANOVA) with Tukey posthoc tests were applied, as appropriate. For the results from TBARS and GPx evaluation, a two-way ANOVA was conducted on the influence of the two independent variables (treatment group, brain hemisphere). The treatment group included G1, G2, G3, and G4, as per Figure 1, and the brain hemisphere consisted of left and right hemispheres, which were evaluated separately. To isolate which groups differed from the other, a multiple comparison procedure was used. The level of significance *p* < 0.05 was accepted. SigmaPlot, v. 11.0 was used for statistical evaluation. All rats survived the 6-OHDA unilateral intrastriatal injection and were included in the statistical analysis.

## 3. Results

### 3.1. Apomorphine Test

**Figure 2:**
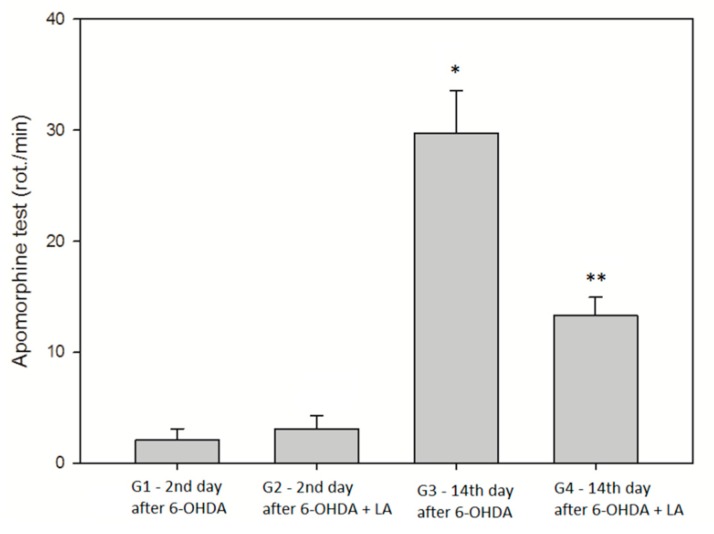
Apomorphine-induced rotation. Data are expressed as mean and standard deviation. G1–G4 are the treatment groups as indicated in Figure 1. * *p* < 0.05 (G3 vs. G1 and G2). ** *p* < 0.05, G4 vs. G1. (Tukey test), *n* = 10 in each group.

The results of the Kruskal–Wallis test were significant (H = 34.041, 3 d.f., (*p* ≤ 0.001)); the mean ranks of rotations per minute were significantly different among the four groups. On day 14, after the 6-OHDA unilateral intrastriatal injection, the apomorphine-induced rotations were statistically significantly higher than those observed on day 2 of the experiment (Figure 2). An increase of the contralateral apomorphine-induced rotations is suggestive of the loss of DA-ergic neurons, a direct toxic effect of the 6-OHDA. Although the tendency for a lower rotation rate in the group G4 vs. G3 was observed, statistical significance could not be reached.

### 3.2. TBARS Evaluation

**Figure 3:**
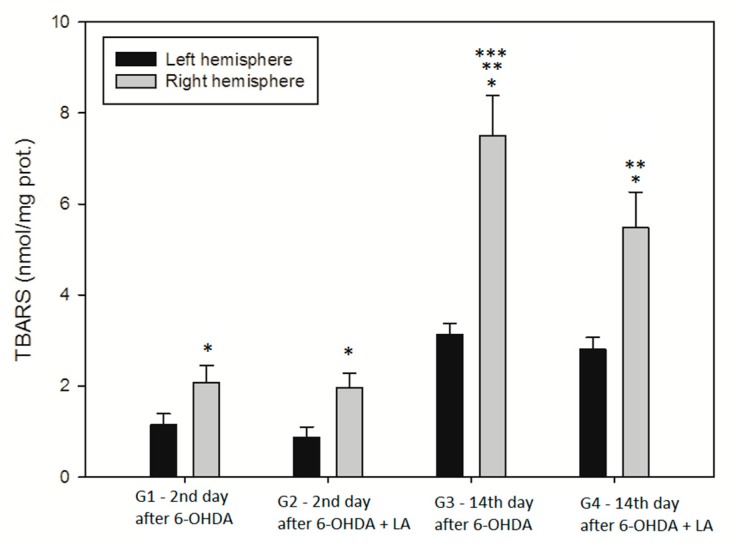
TBARS. Data are expressed as mean and standard deviation. G1, G2, G3, and G4 are the treatment groups as indicated in Figure 1. * *p* < 0.05 (left vs. right hemisphere within each group). ** *p* < 0.05, G3, and G4 vs. G1 and G2. *** *p* < 0.05 G3 vs. G4 (Tukey test), *n* = 10 in each group.

A two-way ANOVA was conducted on the two independent variables (group type, brain hemisphere) on the level of TBARS in the homogenates. All effects were statistically significant at the 0.05 significance levels (Figure 3). The main effect for group type yielded an F ratio of F(3, 72) = 316.836, *p* < 0.001, indicating a significant difference between G1 group (M = 1.613, SD = 0.57), G2 group (M = 1.420, SD = 0.62), G3 group (M = 5.32, SD = 2.33), and G4 group (M = 4.145, SD = 1.48). The main effect for the brain hemisphere yielded an F ratio of F(1. 72) = 443.609, *p* < 0.001, indicated that the effect for the brain hemisphere was significant; left hemisphere (M = 1.989, SD = 1.03), and right hemisphere (M = 4.26, SD = 2.46). The interaction effect was significant; F(3, 72) = 55.633, *p* < 0.001.

Within each group, the TBARS values differed statistically significantly between the left and the right hemispheres, indicating that the 6-OHDA generated higher lipid peroxidation where it was applied compared to the contralateral hemisphere. Statistically significantly higher TBARS levels were found in both groups, evaluated on day 14 of the experiment (G3 and G4), as compared with earlier on day 2 of the experiment (G1 and G2). The TBARS level in the group with LA chronical treatment (G4) was statistically significantly lower than the group without LA treatment (G3).

### 3.3. GPx

**Figure 4:**
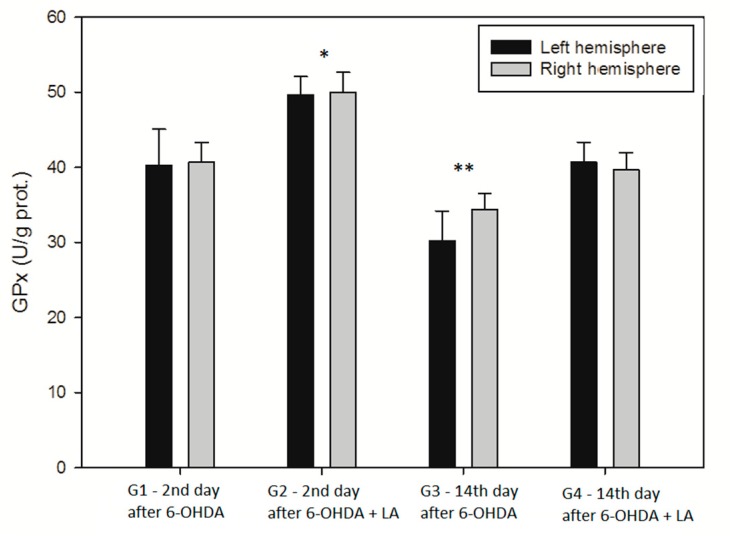
GPx activity. Data are expressed as mean and standard deviation. G1, G2, G3, and G4 are the treatment groups as indicated in Figure 1. * *p* < 0.05, G2 vs. G1, G3, and G4. ** *p* < 0.05, G3 vs. G1, G2 and G4. (Tukey test), *n* = 10 in each group.

A two-way ANOVA was conducted on the two independent variables (group type, brain hemisphere) on the level of GPx activity in the homogenates. All effects were statistically significant at the 0.05 significance levels, except for the brain hemisphere factor (Figure 4). The main effect for group type yielded an F ratio of F(3, 72) = 108.658, *p* < 0.001, indicating a significant difference between G1 group (M = 40.5, SD = 3.76), G2 group (M = 49.8, SD = 2.52), G3 group (M = 32.3, SD = 3.77), and G4 group (M = 40.2, SD = 2.44). The main effect for the brain hemisphere yielded an F ratio of F(1. 72) = 2.121, *p* = 0.15, indicating that the effect for the brain hemisphere was not significant; left hemisphere (M = 40.20, SD = 7.77), and right hemisphere (M = 41.20, SD = 6.15). The interaction effect was not significant, F(3, 72) = 2.645, *p* < 0.056, indicating that the effect of different levels of group type does not depend on what level of brain hemisphere is present.

Statistically significantly higher GPx activity, as compared with the other three groups, was recorded in the group G2, i.e., LA-supplemented and evaluated on day 2 of the experiment. Statistically significantly lower GPx activity, as compared with the other three groups, was recorded in the group G3, i.e., on day 14 of the 6-OHDA application, without LA supplementation.

## 4. Discussion

LA has been subjected to intensive study since the discovery of its antioxidant properties in the 1950s. A lot of in vitro and in vivo preclinical studies have demonstrated potential antioxidant and neuroprotective effects of LA, with a potential benefit for patients with PD. However, the translation of those results into clinical practice has not been done. Despite pathophysiologic, epidemiologic, and mechanistic data suggesting otherwise, clinical trials with antioxidants are mostly negative in the setting of chronic therapy [40]. As far as we know, no one clinical guideline recommends the use of LA by patients with PD. The precise characterization of the place of LA in PD needs randomized clinical trials (RCTs). The well-designed RCTs depend on sufficient and precisely described results from preclinical studies.

Our study was conducted for testing the effect of LA supplementation in 6-OHDA unilateral intrastriatal injected rats. Levodopa (the precursor of DA and treatment of choice for PD), DA itself, and 6-OHDA have a comparable pro-oxidant potential toward neurons. The toxin 6-OHDA is a highly oxidizable DA analog that selectively damages catecholaminergic neurons via the generation of OS. 6-OHDA has been used since 1968 for reproducing several cell processes identified in PD, closely related to neuroinflammation and neuronal death. Its neurotoxicity shares molecular mechanisms similar to the neurotoxicity of DA itself [41]. From previous studies, one knows that 6-OHDA is involved in auto-oxidation, intraneuronal production of reactive oxygen species (ROS), and, via caspase 3-dependent activation of protein kinase Cδ, is able to activate apoptosis [42]. Potent inhibition of the mitochondrial respiratory chain complexes was also observed [43]. Early microglial activation and NADPH oxidase-derived ROS was recently described as a third possible mechanism to contribute to OS generation and 6-OHDA neurotoxicity [44]. An insoluble polymeric pigment related to neuromelanin is finally produced.

From another experiment, it was reported that 6-OHDA induced high levels of OS and strongly upregulated autophagy and neurodegeneration [45]. In our experiment, we demonstrated a statistically significantly elevated level of lipid peroxidation (TBARS), and a decreased GPx activity 14 days after the 6-OHDA, which is suggestive of 6-OHDA toxicity. GPx is susceptible to inactivation by its substrates [46]. This fact could explain why on day 14 of our experiment, we observed significantly lower GPx activity in the 6-OHDA treated rats, which did not receive LA.

The postsynaptic agonist apomorphine induced rotations contralateral to the lesioned sites because of the stimulation of denervation-induced upregulated D2 receptors. It is accepted that the magnitude of nigrostriatal lesions correlates with the circling motor behavior [47]. In our experiment, the contralateral rotation significantly increased on day 14 of the experiment. Typically, unilateral 6-OHDA injection into one hemisphere is used as a hemiparkinsonian model. On the other hand, this is an acute model of neurotoxicity, which is lacking Lewy bodies and the progressive, age-dependent effects of PD [48]. Similar clinical situations could result from a sudden increase in dopamine levels due to the loss of tight dopaminergic regulation. It is known, for example, that in about half of patients, levodopa induces dyskinesias after 5 to 10 years of treatment, for which OS and neuroinflammation are proposed as possible mechanisms. This treatment-related complication in PD is another clinical reason for seeking antioxidation and neuroprotection.

A dose-dependent antidyskinetic effect of LA was recently reported. The likely explanation was its ability to reduce both OS and apoptosis in the neurons [49]. Other beneficial mechanisms are revealed with its ability to correct the metabolism of DA and serotonin to the levels of the intact animals when LA was tested in combination with carnosine as a nanomicellar complex [50]. Serotoninergic neurons are a significant source of DA release in striatal synapses. Their inability to autoregulate in response to elevated DA is a mechanism implicated in levodopa-induced dyskinesia [51]. LA is suggested as a promising disease-modifying therapy that slows the loss of dopaminergic neurons in patients while also delaying the onset of levodopa-induced dyskinesia [52]. Aiming to overcome the OS associated with levodopa therapy, a series of multifunctional hybrid molecules by binding levodopa or DA scaffolds to an LA were synthesized by other investigators [53].

Our study demonstrated that LA (35 mg/kg i.p.) for 14 days statistically significantly increased GPx activity and decreased TBARS in a model of 6-OHDA unilateral intrastriatal injected rats. This effect was in parallel with the lower apomorphine-induced rotation on day 14, as compared with rats, which did not receive LA. However, statistical significance could not be reached. A plausible explanation could be the acute 6-OHDA toxicity effect resulting in maximal neuronal lesions.

Another clinical condition, which is also related to dopamine dysregulation syndrome in patients treated with dopaminergic agonists, is the development of the impulsive/compulsive behavior. This disorder comprises compulsive overuse of medications, gambling, sexual disinhibition, etc. The 6-OHDA rats were recently proposed as a model for studying addiction-like responses in PD by other investigators [54].

Most of the preclinical LA evaluations were performed with doses of 50 or 100 mg/kg p.o. In rotenone-induced damage of the nigral dopaminergic neurons, supplementation with LA 50 mg/kg/day/12 doses p.o. improved motor performance and reduced the level of lipid peroxidation [55]. Our results support the antioxidant effect of LA. In addition, they coincide with similar results reporting that LA 100 mg/kg decreases TBARS production in a rat model of hemi-Parkinsonism by other authors [56]. Toxicological studies from other authors report a NOAEL (no observed adverse effect level) value for rats of 60 mg/kg/day after long-term LA supplementation [57]. The oral LA-resorption is highly variable, so we preferred to use an intraperitoneal application in our experiment. It was demonstrated that even lower doses of LA do have biological activity, i.e., decreasing the blood pressure [58], protection from cyclophosphamide-induced reproductive toxicity [59], or reduction of vascular oxidative stress [60]. Treatment with higher doses, however, displays behavioral and neurological activity in preclinical settings [61]. The long-term utility of LA poses another challenge. Almost all antioxidants can become pro-oxidant, displaying a hermetic dose-response curve. Under this paradigm, lower doses of antioxidants could be beneficial, whereas high doses can be detrimental. In a study on OS in patients with diabetic neuropathy treated with LA, the positive effect lasted up to 60 days. After that, a hermetic effect was observed [62].

Several potential LA-induced mechanisms have been published. LA is an essential cofactor for mitochondrial metabolism. It is required for stabilizing multiple ROS-regulated mitochondrial 2-ketoacid dehydrogenase complexes with essential functions in cell growth, mitochondrial activity, and coordination of fuel metabolism [63]. Besides this, LA is considered as a caloric restriction mimetic compound, due to its depleting effects on the cytosolic acetyl-coenzyme A [64]. LA biological activity mostly relies on the ability to induce autophagy, and this effect was recently reported for in vitro testing of cell lines with 6-OHDA [65]. The reduced form of LA, dihydrolipoic acid, cooperates with rhodanese (EC 2.8.1.1) in sulfane sulfur metabolism and hydrogen sulfide (H2S) release [66]. Hydrogen sulfide increases the substrate for the production of GSH, directly stimulating γ-glutamylcysteine synthase (γ-GCS, EC 6.3.2.2), a rate-limiting enzyme, which regulates the generation of GSH, and also acts as a ROS scavenger [67].

An intriguing new finding has been made on the significant histone deacetylase (HDAC, EC 3.5.1.98) inhibitory activity of LA, allowing epigenetic modification of the nuclear factor erythroid 2-related factor (2Nrf2) [68]. Nrf2 is essential for supporting and maintaining normal mitochondrial function and structural integrity, particularly under conditions of cellular/neuronal stress inherent in neurodegenerative disorders [69]. DNA methylation-dependent modulation of the expression of IL-1β and IL-6 by LA was recently demonstrated in in vitro assays [70]. The emerging role of epigenetics in the mechanism of action of LA paves new directions for study.

## 5. Conclusions

Our results show the beneficial effect of LA in 6-OHDA-induced OS. OS is one of the leading players in the pathophysiology of PD, but is also implicated in long-term treatment-related complications in PD. Our study demonstrates that a low dose of LA could be of value for decreasing the OS of the neurotoxic 6-OHDA, supporting the need for further study of the benefit of LA treatment in PD. OS cannot be considered on its own, as it is intimately connected to autophagy, neuroinflammation, apoptosis, protein misfolding, and numerous changes in the neurotransmitters. Better elucidation of those mechanisms could be used for the development of clinical trials with LA in PD.

## References

[1] Kalia L.V., Lang A.E. (2015). Parkinson’s disease. Lancet.

[2] Tamtaji O.R., Reiter R.J., Alipoor R., Dadgostar E., Kouchaki E., Asemi Z. (2020). Melatonin and Parkinson Disease: Current Status and Future Perspectives for Molecular Mechanisms. Cell. Mol. Neurobiol..

[3] Soto-Otero R., Méndez-Álvarez E., Hermida-Ameijeiras Á., Muñoz-Patiño A.M., Labandeira-Garcia J.L. (2002). Autoxidation and Neurotoxicity of 6-Hydroxydopamine in the Presence of Some Antioxidants: Potential Implication in Relation to the Pathogenesis of Parkinson’s Disease. J. Neurochem..

[4] Quintana-Cabrera R., Fernandez-Fernandez S., Bobo-Jimenez V., Escobar J., Sastre J., Almeida A., Bolaños J.P. (2012). γ-Glutamylcysteine detoxifies reactive oxygen species by acting as glutathione peroxidase-1 cofactor. Nat. Commun..

[5] Yoo S.-E., Chen L., Na R., Liu Y., Rios C., Van Remmen H., Richardson A., Ran Q. (2012). Gpx4 ablation in adult mice results in a lethal phenotype accompanied by neuronal loss in the brain. Free Radic. Biol. Med..

[6] Guiney S.J., Adlard P.A., Bush A.I., Finkelstein D.I., Ayton S. (2017). Ferroptosis and cell death mechanisms in Parkinson’s disease. Neurochem. Int..

[7] Lei X.G., Cheng W.-H., McClung J.P. (2007). Metabolic Regulation and Function of Glutathione Peroxidase-1. Annu. Rev. Nutr..

[8] Ciulla M., Marinelli L., Cacciatore I., Stefano A.D. (2019). Role of Dietary Supplements in the Management of Parkinson’s Disease. Biomolecules.

[9] Spencer J.P.E., Jenner P., Daniel S.E., Lees A.J., Marsden D.C., Halliwell B. (2002). Conjugates of Catecholamines with Cysteine and GSH in Parkinson’s Disease: Possible Mechanisms of Formation Involving Reactive Oxygen Species. J. Neurochem..

[10] Puspita L., Chung S.Y., Shim J. (2017). Oxidative stress and cellular pathologies in Parkinson’s disease. Mol. Brain.

[11] Angelopoulou E., Piperi C., Papavassiliou A.G. (2018). High-mobility group box 1 in Parkinson’s disease: From pathogenesis to therapeutic approaches. J. Neurochem..

[12] Zhu Z., Yang C., Iyaswamy A., Krishnamoorthi S., Sreenivasmurthy S.G., Liu J., Wang Z., Tong B.C.-K., Song J., Lu J. (2019). Balancing mTOR Signaling and Autophagy in the Treatment of Parkinson’s Disease. Int. J. Mol. Sci..

[13] Francisqueti-Ferron F.V., Ferron A.J.T., Garcia J.L., Silva C.C.V.d.A., Costa M.R., Gregolin C.S., Moreto F., Ferreira A.L.A., Minatel I.O., Correa C.R. (2019). Basic Concepts on the Role of Nuclear Factor Erythroid-Derived 2-Like 2 (Nrf2) in Age-Related Diseases. Int. J. Mol. Sci..

[14] Cleeter M.W.J., Chau K.-Y., Gluck C., Mehta A., Hughes D.A., Duchen M., Wood N.W., Hardy J., Mark Cooper J., Schapira A.H. (2013). Glucocerebrosidase inhibition causes mitochondrial dysfunction and free radical damage. Neurochem. Int..

[15] Bae E.-J., Ho D.-H., Park E., Jung J.W., Cho K., Hong J.H., Lee H.-J., Kim K.P., Lee S.-J. (2013). Lipid Peroxidation Product 4-Hydroxy-2-Nonenal Promotes Seeding-Capable Oligomer Formation and Cell-to-Cell Transfer of α-Synuclein. Antioxid. Redox Signal..

[16] Li L., Chen Y., Gibson S.B. (2013). Starvation-induced autophagy is regulated by mitochondrial reactive oxygen species leading to AMPK activation. Cell. Signal..

[17] Jiang S., Fan J., Wang Q., Ju D., Feng M., Li J., Guan Z., An D., Wang X., Ye L. (2016). Diosgenin induces ROS-dependent autophagy and cytotoxicity via mTOR signaling pathway in chronic myeloid leukemia cells. Phytomedicine.

[18] Suzuki M., Bandoski C., Bartlett J.D. (2015). Fluoride induces oxidative damage and SIRT1/autophagy through ROS-mediated JNK signaling. Free Radic. Biol. Med..

[19] Noelker C., Lu L., Höllerhage M., Vulinovic F., Sturn A., Roscher R., Höglinger G.U., Hirsch E.C., Oertel W.H., Alvarez-Fischer D. (2015). Glucocerebrosidase deficiency and mitochondrial impairment in experimental Parkinson disease. J. Neurol. Sci..

[20] Tomassoni D., Amenta F., Di Cesare Mannelli L., Ghelardini C., Nwankwo I.E., Pacini A., Tayebati S.K. (2013). Neuroprotective Activity of Thioctic Acid in Central Nervous System Lesions Consequent to Peripheral Nerve Injury. BioMed. Res. Int..

[21] Shay K.P., Moreau R.F., Smith E.J., Smith A.R., Hagen T.M. (2009). Alpha-lipoic acid as a dietary supplement: Molecular mechanisms and therapeutic potential. Biochim. Biophys. Acta Genl. Subj..

[22] Bjørklund G., Aaseth J., Crisponi G., Rahman M.M., Chirumbolo S. (2019). Insights on alpha-lipoic and dihydrolipoic acids as promising scavengers of oxidative stress and possible chelators in mercury toxicology. J. Inorg. Biochem..

[23] Packer L., Kraemer K., Rimbach G. (2001). Molecular aspects of lipoic acid in the prevention of diabetes complications. Nutrition.

[24] Tibullo D., Li Volti G., Giallongo C., Grasso S., Tomassoni D., Anfuso C.D., Lupo G., Amenta F., Avola R., Bramanti V. (2017). Biochemical and clinical relevance of alpha-lipoic acid: Antioxidant and anti-inflammatory activity, molecular pathways and therapeutic potential. Inflamm. Res..

[25] Phillipson O.T. (2014). Management of the aging risk factor for Parkinson’s disease. Neurobiol. Aging.

[26] Rahimlou M., Asadi M., Banaei Jahromi N., Mansoori A. (2019). Alpha-lipoic acid (ALA) supplementation effect on glycemic and inflammatory biomarkers: A Systematic Review and meta-analysis. Clin. Nutr. ESPEN.

[27] Haghighatdoost F., Hariri M. (2019). The effect of alpha-lipoic acid on inflammatory mediators: A systematic review and meta-analysis of randomized clinical trials. Eur. J. Pharmacol..

[28] Mousavi S.M., Shab-Bidar S., Kord-Varkaneh H., Khorshidi M., Djafarian K. (2019). Effect of alpha-lipoic acid supplementation on lipid profile: A systematic review and meta-analysis of controlled clinical trials. Nutrition.

[29] Han T., Bai J., Liu W., Hu Y. (2012). Therapy of endocrine disease: A systematic review and meta-analysis of α-lipoic acid in the treatment of diabetic peripheral neuropathy. Eur. J. Endocrinol..

[30] Molz P., Schröder N. (2017). Potential Therapeutic Effects of Lipoic Acid on Memory Deficits Related to Aging and Neurodegeneration. Front. Pharmacol..

[31] Zhao H., Zhao X., Liu L., Zhang H., Xuan M., Guo Z., Wang H., Liu C. (2017). Neurochemical effects of the R form of α-lipoic acid and its neuroprotective mechanism in cellular models of Parkinson’s disease. Int. J. Biochem. Cell. Biol..

[32] Zhou L., Cheng Y. (2019). Alpha-lipoic acid alleviated 6-OHDA-induced cell damage by inhibiting AMPK/mTOR mediated autophagy. Neuropharmacology.

[33] De Araújo D.P., Lobato R.D.F.G., Cavalcanti J.R.L.D.P., Sampaio L.R.L., Araújo P.V.P., Silva M.C.C., Neves K.R.T., Fonteles M.M.D.F., Sousa F.C.F.D., Vasconcelos S.M.M. (2011). The Contributions of Antioxidant Activity of Lipoic Acid in Reducing Neurogenerative Progression of Parkinson’s Disease: A Review. Int. J. Neurosci..

[34] Seaton T.A., Jenner P., Marsden C.D. (1996). Thioctic acid does not restore glutathione levels or protect against the potentiation of 6-hydroxydopamine toxicity induced by glutathione depletion in rat brain. J. Neural Transmission.

[35] Paxinos G., Watson C. (2007). The Rat Brain in Stereotaxic Coordinates.

[36] Hudson J.L., Fong C.-S., Boyson S.J., Hoffer B.J. (1994). Conditioned apomorphine-induced turning in 6-OHDA-lesioned rats. Pharmacol. Biochem. Behav..

[37] Flohé L., Günzler W.A. (1984). Assays of glutathione peroxidase. Methods Enzymolog..

[38] Ohkawa H., Ohishi N., Yagi K. (1979). Assay for lipid peroxides in animal tissues by the thiobarbituric acid reaction. Anal. Biochem..

[39] Lowry O.H., Rosenbrough N.J., Farr A., Randall R.J. (1951). Protein measurement with the Folin phenol reagent. J. Biol. Chem..

[40] Steinhubl S.R. (2008). Why Have Antioxidants Failed in Clinical Trials?. Am. J. Cardiol..

[41] Filloux F., Townsend J.J. (1993). Pre- and postsynaptic neurotoxic effects of dopamine demonstrated by intrastriatal injection. Exp. Neurol..

[42] Hanrott K., Gudmunsen L., O’Neill M.J., Wonnacott S. (2006). 6-Hydroxydopamine-induced Apoptosis Is Mediated via Extracellular Auto-oxidation and Caspase 3-dependent Activation of Protein Kinase Cδ. J. Biol. Chem..

[43] Glinka Y., Gassen M., Youdim M.B.H., Riederer P., Calne D.B., Horowski R., Mizuno Y., Poewe W., Youdim M.B.H. (1997). Mechanism of 6-hydroxydopamine neurotoxicity. Advances in Research on Neurodegeneration.

[44] Rodriguez-Pallares J., Parga J.A., Muñoz A., Rey P., Guerra M.J., Labandeira-Garcia J.L. (2007). Mechanism of 6-hydroxydopamine neurotoxicity: The role of NADPH oxidase and microglial activation in 6-hydroxydopamine-induced degeneration of dopaminergic neurons. J. Neurochem..

[45] Marin C., Aguilar E. (2011). In vivo 6-OHDA-induced neurodegeneration and nigral autophagic markers expression. Neurochem. Int..

[46] Cho C.-S., Lee S., Lee G.T., Woo H.A., Choi E.-J., Rhee S.G. (2010). Irreversible Inactivation of Glutathione Peroxidase 1 and Reversible Inactivation of Peroxiredoxin II by H_2_O_2_ in Red Blood Cells. Antioxid. Redox Signal..

[47] Deumens R., Blokland A., Prickaerts J. (2002). Modeling Parkinson’s Disease in Rats: An Evaluation of 6-OHDA Lesions of the Nigrostriatal Pathway. Exp. Neurol..

[48] Tieu K. (2011). A Guide to Neurotoxic Animal Models of Parkinson’s Disease. Cold Spring Harb Perspect. Med..

[49] Zhang S., Xie C., Lin J., Wang M., Wang X., Liu Z. (2018). Lipoic acid alleviates L-DOPA-induced dyskinesia in 6-OHDA parkinsonian rats via anti-oxidative stress. Mol. Med. Rep..

[50] Kulikova O.I., Berezhnoy D.S., Stvolinsky S.L., Lopachev A.V., Orlova V.S., Fedorova T.N. (2018). Neuroprotective effect of the carnosine–α-lipoic acid nanomicellar complex in a model of early-stage Parkinson’s disease. Regul. Toxicol. Pharmacol..

[51] Carta M., Björklund A. (2018). The serotonergic system in L-DOPA-induced dyskinesia: Pre-clinical evidence and clinical perspective. J. Neural. Transm (Vienna).

[52] Martini M.L., Neifert S.N., Mocco J., Panov F., Tse W., Walker R.H., Jin J., Gupta F. (2019). Recent Advances in the Development of Experimental Therapeutics for Levodopa-Induced Dyskinesia. J. Mov. Disord..

[53] Di Stefano A., Sozio P., Cocco A., Iannitelli A., Santucci E., Costa M., Pecci L., Nasuti C., Cantalamessa F., Pinnen F. (2006). L-Dopa− and Dopamine−(*R*)-α-Lipoic Acid Conjugates as Multifunctional Codrugs with Antioxidant Properties. J. Med. Chem..

[54] Campbell J.C., Jeyamohan S.B., Cruz P.D.L., Chen N., Shin D., Pilitsis J.G. (2014). Place conditioning to apomorphine in rat models of Parkinson’s disease: Differences by dose and side-effect expression. Behav. Brain Res..

[55] Zaitone S.A., Abo-Elmatty D.M., Shaalan A.A. (2012). Acetyl-l-carnitine and α-lipoic acid affect rotenone-induced damage in nigral dopaminergic neurons of rat brain, implication for Parkinson’s disease therapy. Pharmacol. Biochem. Behav..

[56] Jalali-Nadoushan M., Roghani M. (2013). Alpha-lipoic acid protects against 6-hydroxydopamine-induced neurotoxicity in a rat model of hemi-parkinsonism. Brain Res..

[57] Cremer D.R., Rabeler R., Roberts A., Lynch B. (2006). Long-term safety of α-lipoic acid (ALA) consumption: A 2-year study. Regul. Toxicol. Pharmacol..

[58] Dudek M., Razny K., Bilska-Wilkosz A., Iciek M., Sapa J., Wlodek L., Filipek B. (2016). Hypotensive effect of alpha-lipoic acid after a single administration in rats. Anatol. J. Cardiol..

[59] Selvakumar E., Prahalathan C., Mythili Y., Varalakshmi P. (2004). Protective effect of dl-α-lipoic acid in cyclophosphamide induced oxidative injury in rat testis. Reprod. Toxicol..

[60] Xiang W., Wang L., Cheng S., Zhou Y., Ma L. (2019). Protective Effects of α-Lipoic Acid on Vascular Oxidative Stress in Rats with Hyperuricemia. Curr. Med. Sci..

[61] De Araújo D.P., De Sousa C.N.S., Araújo P.V.P., Menezes C.E.d.S., Sousa Rodrigues F.T., Escudeiro S.S., Lima N.B.C., Patrocínio M.C.A., Aguiar L.M.V., Viana G.S.d.B. (2013). Behavioral and Neurochemical Effects of Alpha-Lipoic Acid in the Model of Parkinson’s Disease Induced by Unilateral Stereotaxic Injection of 6-Ohda in Rat. Evid. Based Complement. Alternat. Med..

[62] Mrakic-Sposta S., Vezzoli A., Maderna L., Gregorini F., Montorsi M., Moretti S., Greco F., Cova E., Gussoni M. (2018). R(+)-Thioctic Acid Effects on Oxidative Stress and Peripheral Neuropathy in Type II Diabetic Patients: Preliminary Results by Electron Paramagnetic Resonance and Electroneurography. Oxid. Med. Cell. Longev..

[63] Solmonson A., DeBerardinis R.J. (2018). Lipoic acid metabolism and mitochondrial redox regulation. J. Biol. Chem..

[64] Le Noci V., Sommariva M., Bianchi F., Triulzi T., Tagliabue E., Balsari A., Sfondrini L. (2019). Local Administration of Caloric Restriction Mimetics to Promote the Immune Control of Lung Metastases. J. Immunol. Res..

[65] Hu X., Weng Z., Chu C.T., Zhang L., Cao G., Gao Y., Signore A., Zhu J., Hastings T., Greenamyre J.T. (2011). Peroxiredoxin-2 Protects against 6-Hydroxydopamine-Induced Dopaminergic Neurodegeneration via Attenuation of the Apoptosis Signal-Regulating Kinase (ASK1) Signaling *Cascade*. J. Neurosci..

[66] Bilska-Wilkosz A., Iciek M., Kowalczyk-Pachel D., Górny M., Sokołowska-Jeżewicz M., Włodek L. (2017). Lipoic Acid as a Possible Pharmacological Source of Hydrogen Sulfide/Sulfane Sulfur. Molecules.

[67] Kimura Y., Goto Y.-I., Kimura H. (2010). Hydrogen Sulfide Increases Glutathione Production and Suppresses Oxidative Stress in Mitochondria. Antioxid. Redox Signal..

[68] Moos W.H., Faller D.V., Glavas I.P., Harpp D.N., Irwin M.H., Kanara I., Pinkert C.A., Powers W.R., Steliou K., Vavvas D.G. (2017). Epigenetic Treatment of Neurodegenerative Ophthalmic Disorders: An Eye Toward the Future. BioRes. Open Access.

[69] Irwin M.H., Moos W.H., Faller D.V., Steliou K., Pinkert C.A. (2016). Epigenetic Treatment of Neurodegenerative Disorders: Alzheimer and Parkinson Diseases. Drug Dev. Res..

[70] Dinicola S., Proietti S., Cucina A., Bizzarri M., Fuso A. (2017). Alpha-Lipoic Acid Downregulates IL-1β and IL-6 by DNA Hypermethylation in SK-N-BE Neuroblastoma Cells. Antioxidants.

